# N-Acetylglucosamine Induces White to Opaque Switching, a Mating Prerequisite in *Candida albicans*

**DOI:** 10.1371/journal.ppat.1000806

**Published:** 2010-03-12

**Authors:** Guanghua Huang, Song Yi, Nidhi Sahni, Karla J. Daniels, Thyagarajan Srikantha, David R. Soll

**Affiliations:** Department of Biology, The University of Iowa, Iowa City, Iowa, United States of America; University of Wisconsin-Madison, United States of America

## Abstract

To mate, the fungal pathogen *Candida albicans* must undergo homozygosis at the mating-type locus and then switch from the white to opaque phenotype. Paradoxically, opaque cells were found to be unstable at physiological temperature, suggesting that mating had little chance of occurring in the host, the main niche of *C. albicans*. Recently, however, it was demonstrated that high levels of CO_2_, equivalent to those found in the host gastrointestinal tract and select tissues, induced the white to opaque switch at physiological temperature, providing a possible resolution to the paradox. Here, we demonstrate that a second signal, N-acetylglucosamine (GlcNAc), a monosaccharide produced primarily by gastrointestinal tract bacteria, also serves as a potent inducer of white to opaque switching and functions primarily through the Ras1/cAMP pathway and phosphorylated Wor1, the gene product of the master switch locus. Our results therefore suggest that signals produced by bacterial co-members of the gastrointestinal tract microbiota regulate switching and therefore mating of *C. albicans*.

## Author Summary

To mate, the human fungal pathogen *Candida albicans* must undergo a complex phenotypic change from a round “white” to large, elongated “opaque” cell. This involves the regulation of approximately 5% of the organism's genes. Surprisingly, this complex transition is not required for mating in other related yeast. Even more surprisingly, it was found that *in vitro* the mating-competent opaque phenotype was unstable at 37°C, the temperature of the host body. This observation led to a paradox. If *C. albicans* lives primarily in an animal host, physiological temperature would thwart mating, so where does *C. albicans* mate? This led to the suggestion that some physiological condition in the host niche stabilizes the opaque phenotype or even induces switching from white to opaque, so cells can mate. Recently, we demonstrated that the high concentrations of CO_2_ found in tissue and the gastrointestinal tract induced switching from white to opaque and then stabilized the opaque phenotype. Here, we demonstrate that a second factor, N-acetylglucosamine (GlcNAc), a sugar released primarily by bacteria in the gastrointestinal tract, also induces the switch from white to opaque and stabilizes the opaque phenotype. We demonstrate by mutational analysis that GlcNAc induction is regulated primarily by the Ras1/cAMP pathway, which also regulates filamentation of *C. albicans*. This is perhaps not surprising given that white-opaque switching shares with filamentation several phenotypic characteristics. Finally, we show that induction by GlcNAc requires the phosphorylated master switch gene that regulates spontaneous switching, suggesting that it induces the switch from white to opaque by activating the gene product of the master switch gene. Together, our results suggest that multiple signals from bacterial co-members of the gastrointestinal tract microbiota regulate switching and, therefore, mating of *C. albicans* in the colonized host.

## Introduction

The white-opaque transition in MTL-homozygous strains of *Candida albicans* affects cellular physiology, cell morphology, gene expression, virulence and biofilm formation [1]–[3]. It is repressed by the **a**1-α2 co-repressor in **a**/α cells and derepressed in cells that have undergone *MTL*-homozygosis to become either **a**/**a** or α/α [4]. White-opaque switching, which occurs spontaneously and reversibly, is controlled through expression of a master switch locus, *WOR1*, which also has been referred to as *TOS9* [5]–[7]. The frequency of switching is regulated in part at the level of *WOR1* transcription by a number of genes through a network of positive and negative regulatory loops [8],[9] and through changes in chromatin state [10]–[12].

After the discovery of a mating system in *C. albicans* [13], it was demonstrated that *MTL*-homozygous cells had to switch from white to opaque in order to mate [4],[14]. Paradoxically, it was demonstrated that *in vitro* this switch was sensitive to physiological temperature [15],[16]. When the temperature of opaque cell cultures grown at 25°C was raised to 37°C, cells switched *en masse* and semi-synchronously to white [17], suggesting that the opaque phenotype was unstable at physiological temperatures and that mating would, therefore, be compromised in a host, the major niche of *C. albicans*. Recently, we demonstrated that high levels of CO_2_ comparable to those found in the host gastrointestinal tract and some host tissues induced switching from white to opaque, maintained cells in the opaque phenotype, and blocked switching from opaque to white [18]. CO_2_ had been demonstrated previously to be a potent inducer of filamentation as well [19],[20]. Because N-acetylglucosamine (GlcNAc), which is produced by bacteria in the gastrointestinal tract [21], is also a potent inducer of filamentation [22], we therefore considered the possibility that it, like CO_2_, was an inducer of the white to opaque transition.

We found that G1cNAc represents a second strong inducer of the white to opaque transition and stabilizes the opaque phenotype. GlcNAc induction occurs at 25°C and is enhanced at 37°C. In addition, because there were indications that the induction of filamentation by GlcNAc was mediated by the Ras1/cAMP pathway [23]–[30], we tested whether G1cNAc induction of switching was regulated by this pathway. Our results demonstrate that GlcNAc induction is transduced primarily by the same Ras1/cAMP pathway that has been implicated in the regulation of filamentation and requires phosphorylated Wor1, the product of the master switch locus. We therefore suggest that two different signals in the host gastrointestinal tract, both produced by bacterial co-members of the gastrointestinal tract microbiota, can regulate the white to opaque transition, an essential step in *C. albicans* mating.

## Results

### GlcNAc Induction of Switching

**Figure 1:**
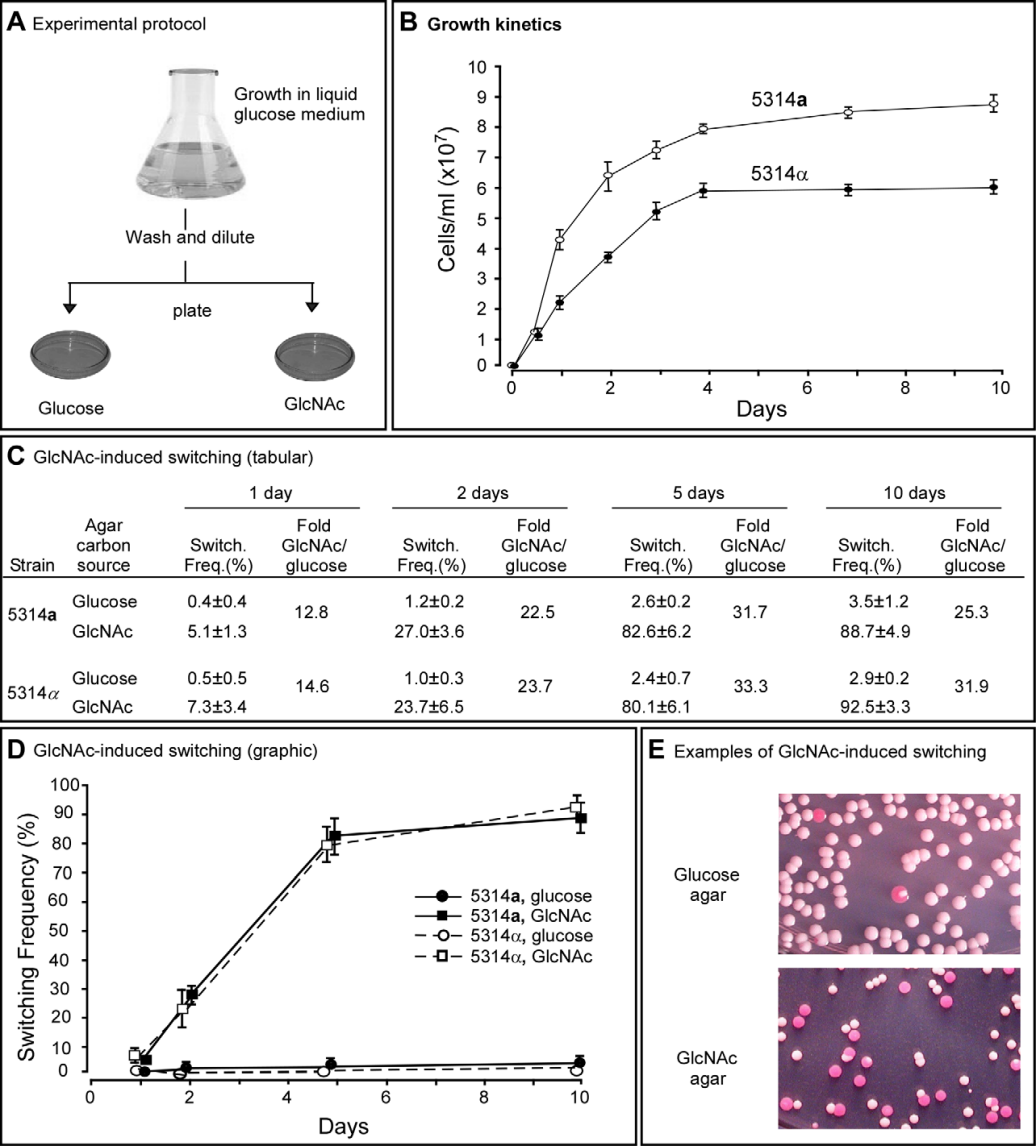
GlcNAc induces switching from white to opaque in a and α cells of *Candida albicans*. A. The experimental protocol for assessing GlcNAC induction. Cells are first grown in liquid glucose medium at 25°C and then plated on glucose or GlcNAc agar at 25°C and in select cases at 37°C. B. Growth kinetics of **a** and α cells in liquid glucose medium at 25°C. C. Switching frequencies (Switch. Freq.) on glucose or GlcNAc agar of cells grown at 25°C in liquid glucose medium for 1, 2, 5 and 10 days. The colony number assessed for phenotype varied between 370 and 700 under the varied conditions. D. A graph of the switching frequencies from panel C. E. Examples of 3 day cultures on glucose or GlcNAc agar at 25°C. Switching frequencies were measured after 5 days on agar. No. Cols., number of colonies.

To test whether GlcNAc induces the white to opaque transition and does so as a function of culture age, as is the case for the induction of filamentation [22],[24], white cells of **a**/**a** and α/α derivatives of strain SC5314, 5314**a** and 5314α, respectively, were first grown at 25°C in suspension in liquid modified Lee's medium in which glucose was the sole carbon source (“liquid glucose medium”) [31] (Figure 1A). To assess GlcNAc induction as a function of culture growth [23], cells were removed at time intervals from the liquid culture, plated on nutrient agar containing either 1.25% (w/v) glucose (“glucose agar”) or 1.25% (w/v) GlcNAc (“GlcNAc agar”) as the sole carbon source (Figure 1A), and incubated at 25°C. This temperature was selected to assess induction initially, because physiological temperature (37°C) induces the reverse switch from opaque to white [15],[17], and we wanted the initial assessment to be performed in the absence of reverse induction. After five days on agar, the proportion of opaque colonies plus white colonies with opaque sectors was measured in glucose or GlcNAc agar. This proportion will be referred to as the “switching frequency” for convenience, but should not be confused with the rate of switching [1],[16],[32]. Although **a**/**a** and α/α cultures reached different final cell densities, they entered the saturation phase in liquid glucose medium at approximately the same time (Figure 1B).

For **a**/**a** cells plated on glucose agar at 25°C, the switching frequency increased from 0.4±0.4% for cells taken from exponential phase cultures after one day, to 3.5±1.2% for cells taken from late saturation phase cultures after 10 days (Figure 1C, D). For α/α cells, the proportion increased similarly from 0.5±0.5% to 2.9±0.2% (Figure 1C, D). Hence, the switching frequency of **a**/**a** and α/α cells grown in liquid glucose medium increased 9.5- and 6.0-fold, respectively, over the course of exponential growth and entrance into the saturation phase. For **a**/**a** cells plated on GlcNAc agar, the frequency of switching increased from 5.1±1.3% after one day to 88.7±4.9% after 10 days, and for α/α cells, the frequency increased from 7.3±3.4% to 92.5±3.3% (Figure 1C, D). Plating on GlcNAc agar, therefore, caused an increase in the frequency of switching of **a**/**a** and α/α cells after one day that was approximately 13- and 15-fold higher, respectively, than the frequencies on glucose agar after one day (Figure 1C, D). After 10 days, the frequency was 25- and 32-fold higher, respectively, than the frequency on glucose agar (Figure 1C, D). In Figure 1E, examples are presented of cultures from three day liquid glucose cultures of **a**/**a** cells plated on glucose agar or GlcNAc agar. Note that on GlcNAc agar, the majority of colonies were completely opaque rather than sectored, indicating that in these cases GlcNAc induction occurred very early in the life history of the colonies. Similar results were obtained for cells grown in liquid glucose medium for five days and plated on agar containing either glucose or GlcNAc ranging in concentration from 0.2% to 5% (w/v), indicating that the concentration employed (1.25%, w/v) resulted in maximum G1cNAc induction (data not shown).

### GlcNAc Induction at 37°C

The preceding experiments were performed at 25°C. To test whether GlcNAc also induced white to opaque switching at physiological temperature (37°C), we then performed experiments in which white cells of **a**/**a** and α/α derivatives of strain SC5314 were grown to mid-log phase on liquid glucose medium for 48 hr at 25°C, then plated on glucose or GlcNAc agar at either 25 or 37°C. Increasing the temperature from 25 to 37°C on glucose agar resulted in a lower frequency of switching for white cells of both the **a**/**a** and α/α strains (Table 1, data in air). In direct contrast, when white cells of both strains were grown in liquid glucose medium at 25°C and then plated on GlcNAc agar at 37°C, there was a dramatic increase in the switching frequency (Table 1, data in air). These data demonstrate that physiological temperature enhances GlcNAc induction.

**Table 1:** GlcNAc induction of white-to-opaque switching is enhanced at 37°C and in 1% CO_2._

Strain	Agar Medium, Temp	Air	1% CO_2_
		Switching frequency (%)	Switching frequency (%)
5314a	Glucose, 25°C	0.4±0.6	21.4±1.4
	37°C	<0.4	7.5±1.5
	GlcNAc, 25°C	26.7±1.2	100.0±0.0
	37°C	98.9±1.2	100.0±0.0
5314α	Glucose, 25°C	0.2±0.4	19.4±1.8
	37°C	<0.2	7.1±0.5
	GlcNAc, 25°C	22.4±3.3	100.0±0.0
	37°C	99.7±0.6	99.4±0.5

As described in Figure 1A, white cells were cultured for 48 hours in glucose liquid medium at 25°C, then plated onto glucose or GlcNAc agar, and incubated at 25 or 37°C in air or air containing 1% CO_2_. Total colonies analyzed in each strain and condition varied between 150 and 400.

### The Role of Ras1

**Figure 2:**
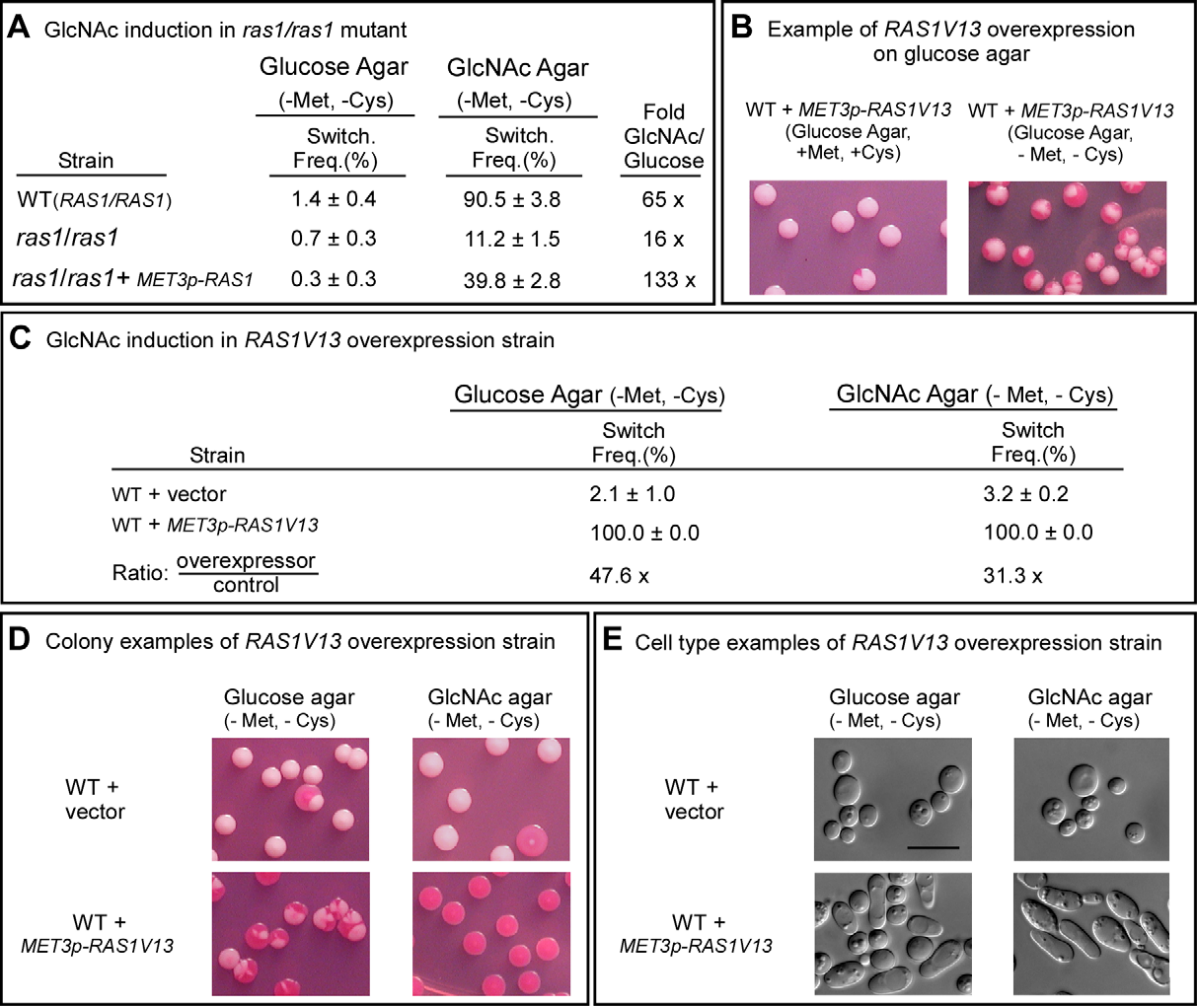
Ras1 plays a major role in GlcNAc induction. A. Switching frequencies (Switch. Freq.) of wild type (WT) and *ras1/ras1* cells grown in a glucose liquid medium at 25°C for 7 days and then plated on glucose agar and GlcNAc agar at 25°C. The colony numbers assessed for phenotype varied between 600 and 1,000 under varied conditions. B. Colonies of the inducible strain WT+*MET3p*-*RAS1V13* grown on glucose at 25°C agar in the presence (repressing condition) or absence (activating condition) of methionine and cysteine. White cells from glucose agar (repressing condition) were plated. C. Switching frequencies of cells of the control strain WT+vector and the *RAS1V13* overexpression strain WT+*MET3p*-*RAS1V13* grown at 25°C in glucose liquid medium for one day and then plated on glucose or GlcNAc agar at 25°C. The colony numbers assessed ranged between 250 and 400. D. Examples of colonies on glucose and GlcNAc agar under activating conditions. E. Examples of cells from colonies on glucose and GlcNAc agar at 25°C under activating conditions. The scale bars in panel E represents 10 µm. Switching frequencies were measured after 5 days of growth at 25°C on agar.

The cAMP pathway, which plays a role in filamentation, includes Ras1, Cdc35, Pde2 and the protein kinase isoforms Tpk1 and Tpk2 [25]–[30]. To test whether GlcNAc induction of white to opaque switching was mediated by the cAMP pathway, we first analyzed the *RAS1* deletion mutant, *ras1/ras1*. These experiments were performed at 25°C because a temperature of 37°C induced a portion of the cells of mutants of the Ras1/cAMP pathway to undergo filamentation (data not shown), making it difficult to assess switching from white to opaque at the level of cell phenotype. White cells of *ras1/ras1* and the control strain (WT) were grown at 25°C in liquid glucose medium to saturation phase (seven days), plated on either glucose or GlcNAc agar, and analyzed for switching frequencies after five days at 25°C. The switching frequency on GlcNAc agar was 90.5±3.8% for WT cells, and 11.2±1.5% for *ras1/ras1* cells (Figure 2A), indicating that Ras1 played a major, but not exclusive, role in GlcNAc induction. The frequency of switching of *ras1/ras1* cells on GlcNAc agar was 9-fold lower than that of WT cells, and 16-fold higher than that on glucose agar (Figure 2A). Complementation of *ras1/ras1* with *RAS1* under the control of the *MET3* promoter partially rescued the mutant phenotype in the activated state (Figure 2A). Rescue was incomplete due to the fact that *RAS1* was controlled in the complemented strain by the *MET3* rather than the natural promoter [7],[33]. It should also be noted that on glucose agar, the frequency of switching of WT cells was two-fold higher than that of *ras1/ras1* cells (Figure 2A), indicating that a *RAS1*-dependent pathway also played a role in spontaneous switching on glucose agar.

To explore further the role of *RAS1* in G1cNAc induction, we transformed strain WUM5A, a derivative of α/α strain WO-1, with *RAS1V13*, which encodes a constitutively activated form of Ras1 (Ras1V13 [25]) under the control of the *MET3* promoter *MET3p* [33], to generate strain WT+*MET3p*-*RAS1V13*. The control strain was also transformed with the vector lacking the *RAS1V13* to generate the control strain WT+vector. The addition of 2.5 mM methionine plus 2.5 mM cysteine (+Met, +Cys) represses *MET3* promoter activity (the repressed state) and the absence (-Met, -Cys) activates it (the activated state) [33]. White cells of WT+*METp*-*RAS1V13* and WT+vector were plated directly onto glucose agar in the presence or absence of methionine and cysteine at 25°C. In the repressed state (+Met, +Cys), the majority of colonies were white, with few sectors, but in the activated state (-Met, -Cys), nearly every colony was highly sectored (Figure 2B), indicating that overexpression of *RAS1V13* in the absence of GlcNAc induced switching. Next, white cells of strains WT+vector and WT+*METp*-*RAS1V13* were grown in liquid glucose medium in the repressed state for one day to the mid-exponential phase, then plated on either glucose or GlcNAc agar in the induced state. On glucose agar, the switching frequency of white WT+vector cells was 2.1±1.0%, and for the overexpression strain, 100% (Figure 2C). The majority of colonies of the overexpression mutant on glucose agar were highly sectored white colonies (Figure 2D). Only 2.3±1.2% were homogeneous opaque colonies. On GlcNAc agar, the switching frequency of control cells was 3.2±0.2%, while that of the overexpression mutant was 100% (Figure 2C). All of the latter colonies were homogeneously opaque (Figure 2D). The near uniformity of the opaque phenotype in the latter colonies was evident at the cellular level (Figure 2E). These results reinforce the conclusion that induction of white to opaque switching by GlcNAc is mediated primarily by Ras1.

### The Role of cAMP

**Figure 3:**
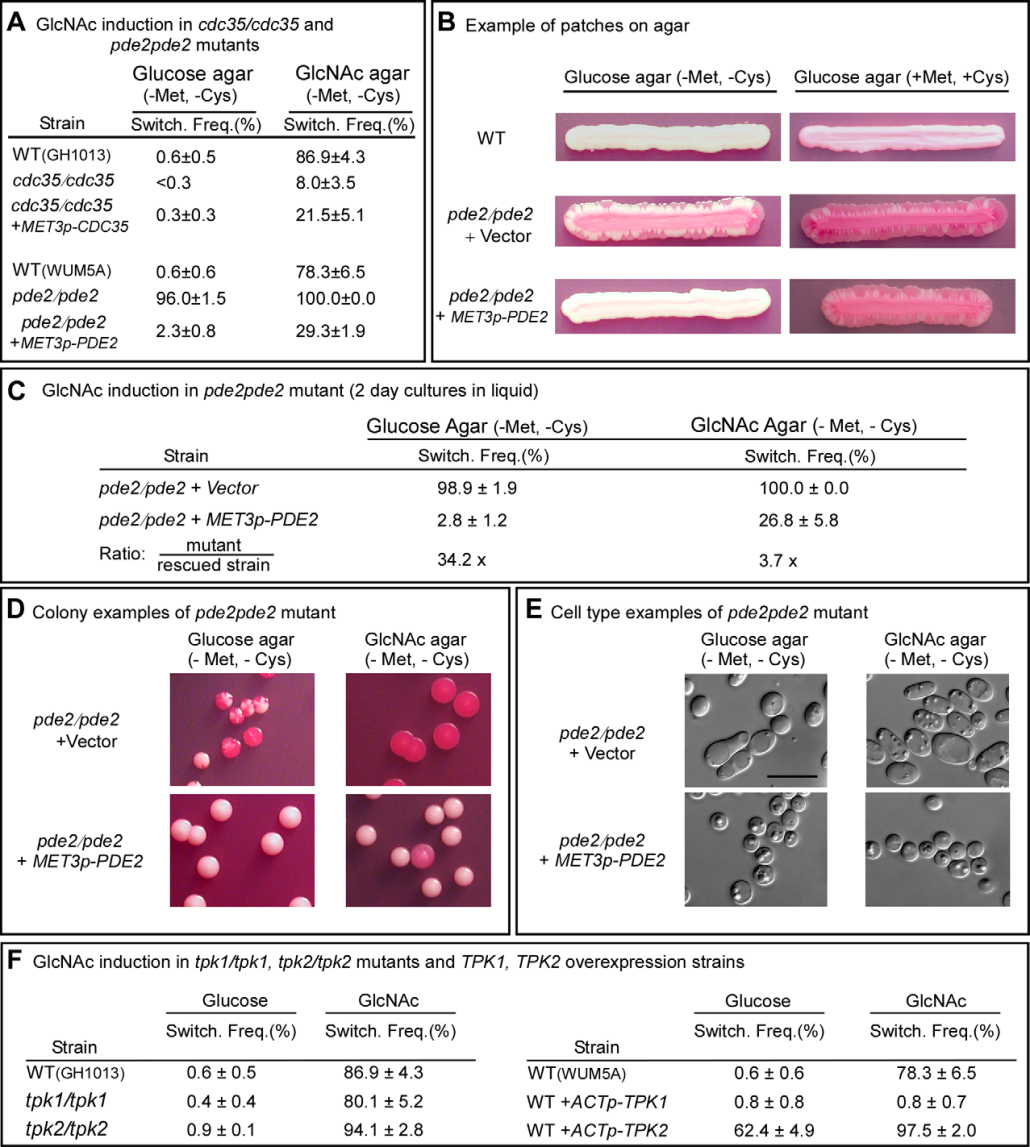
The genes *CDC35*, *PDE2*, *TPK1* and *TPK2* play roles in GlcNAc induction of switching. A. Switching frequencies (Switch. Freq.) of cells of the mutants *cdc35/cdc35* and *pde2/pde2* grown at 25°C in glucose liquid medium for 7 days (*cdc35/cdc35*) or 5 days (*pde2/pde2*), and then plated on glucose or GlcNAc agar at 25°C. The number of assessed colonies ranged between 200 and 450. B. Streaks of cells grown for 5 days of the parental control strain (WT), the mutant control strain *pde2/pde2*+vector and the inducible strain *pde2/pde2*+*MET3p*-*PDE2* under inducing (-Met, -Cyc) and inducing (+Met, +Cys) condition. C. Switching frequencies of cells of the mutant control *pde2/pde2*+vector and the rescued strain *pde2/pde2*+*MET3p*-*PDE2* grown in liquid glucose medium for 2 days and then plated on glucose and GlcNAc agar under activating conditions. The number of assessed colonies ranged between 350 and 450. D. Examples of colonies of *pde2/pde2*+vector and *pde2/pde2*+*MET3p*-*PDE2* from panel C under activating conditions. E. Examples of cells from colonies in Figure D. F. Switching frequencies of parental control strains, the mutants *tpk1/tpk1* and *tpk2/tpk2* and the overexpression strains WT+*ACTp*-*TPK1* and WT+*ACTp*-*TPK2* grown in glucose medium for 5 days and then plated on glucose or GlcNAc agar. The number of assessed colonies ranged between 250 and 800 colonies.

In the cAMP pathway that is involved in filamentation, Ras1 activates adenylate cyclase, which is encoded by *CDC35* [26]. The resulting increase in cAMP is kept in check by a cAMP-phosphodiesterase, which is encoded by *PDE2* [34],[35]. If GlcNAc induction of white-opaque switching is mediated by the same cAMP pathway, then deletion of *CDC35* should reduce the effect, while deletion of *PDE2* should enhance it. When white *cdc35/cdc35* cells were grown at 25°C to saturation phase in liquid glucose medium (seven days) and then plated on GlcNAc agar, the frequency of switching was 8.0±3.5%, whereas that of the WT parental control was 86.9±4.3% (Figure 3A). Complementation of the mutant *cdc35/cdc35* with *CDC35* under control of the *MET3* promoter partially rescued the mutant phenotype (Figure 3A). These results indicate that *CDC35* is necessary for the major response to GlcNAc, as was the case for *RAS1*. GlcNAc did, however, induce low level switching in white *cdc35/cdc35* cells, indicating that although *CDC35* is necessary for the major response to GlcNAc, there is a minor response that is *CDC35*-independent, just as we observed there is a minor response that is *RAS1*-independent.

When white *pde2/pde2* cells were grown at 25°C to saturation phase in liquid glucose medium (five days) and then plated on GlcNAc agar, the switching frequency was 100%, compared to 78.6±6.5% for control cells (Figure 3A). When plated on glucose agar, the frequency of switching was 96.0±1.5%, compared to 0.6±0.6 for WT cells (Figure 3A). Complementation of the *pde2/pde2* mutant with *PDE2* under the control of the *MET3* promoter partially rescued the mutant phenotype (Figure 3A).

To explore further the role of Pde2 in switching, the deletion mutant *pde2/pde2* was also transformed with a vector containing *PDE2* under the control of the *MET3* promoter to generate the strain *pde2/pde2*+*MET3p*-*PDE2*. The deletion mutant *pde2/pde2* was transformed with the vector lacking *PDE2* to generate the control strain *pde2/pde2*+vector. When white cells of the parental control (WT) were grown at 25°C as a streak on glucose agar lacking methionine and cysteine (activating conditions), only rare opaque sectors formed at the periphery (Figure 3B). When the mutant *pde2/pde2*+vector was streaked at 25°C, opaque sectors rimmed the entire streak (Figure 3B). In contrast, opaque sectors were absent at the periphery of the streak of the overexpression mutant *pde2/pde2*+*MET3p*-*PDE* under activating conditions (Figure 3B). When the overexpression mutant *pde2/pde2*+*MET3p*-*PDE* was grown at 25°C in liquid glucose medium under activating conditions to early exponential phase (two days), then plated on glucose agar at 25°C under activating conditions, the frequency of sectoring was 2.8±1.2%, compared to 98.9±1.9% for the deletion mutant (Figure 3C). When plated at 25°C on GlcNAc agar under activating conditions, the frequency of switching of the rescued strain was 26.8±5.8% compared to 100% for the mutant (Figure 3C). Examples of colonies of strain *pde2/pde2*+vector and *pde2/pde2*+*MET3p*-*PDE* grown at 25°C on glucose or GlcNAc agar under activating conditions are presented in Figure 3D, and examples of cells from these colonies are presented in Figure 3E. These results support the conclusion that cAMP is involved in the regulation of the GlcNAc response and that Pde2 plays the role of a negative regular.

### The Role of the Protein Kinases

In the Ras1/cAMP pathway, cAMP activates protein kinase A (PKA) [27],[36]. In *S. cerevisiae* there are three PKA catalytic subunits, Tpk1, Tpk2 and Tpk3, which play roles in the cAMP pathway regulating pseudohypha formation [36],[37]. *C. albicans* possesses two isoforms, Tpk1 and Tpk2, which have been demonstrated to play functionally different roles in filamentation, depending upon environmental conditions [27],[38]. Consistent with previous reports [27], our lack of success in generating a double mutant of *TPK1* and *TPK2* suggested that this mutant may not be viable. We therefore analyzed the individual deletion mutants *tpk1/tpk1* and *tpk2/tpk2*. White cells of the individual deletion mutants were grown at 25°C in liquid glucose medium to saturation phase (seven days) and then plated on glucose or GlcNAc agar and examined for switching after five days. Deletion of either *TPK1* or *TPK2* had no detectable effect on the frequency of switching on glucose or on GlcNAc agar (Figure 3F). There was, however, one noticeable difference in the GlcNAc-induced opaque colonies of *tpk1/tpk1*. They possessed a mixture of opaque cells and cells that had formed hyphae, suggesting that Tpk1 plays a role in the regulation of the bud-hyphae transition (data not shown).

Given that each of the alternative PKA isoforms may perform redundant function in the two mutants *tpk1/tpk1* or *tpk2/tpk2*, we generated overexpression mutants in the wild type background WUM5A (WT) in which *TPK1* or *TPK2* was placed under the regulation of the strong constitutive *ACT1* promoter [5]. White cells of the overexpression strains WT+*ACTp*-*TPK1* and WT+*ACTp*-*TPK2*, as well as white cells of the control strain, were grown for five days at 25°C to saturation phase in liquid glucose medium and then plated on glucose or GlcNAc agar and examined after five days at 25°C for switching (Figure 1A). Overexpression of *TPK1* had no effect on the switching frequency on glucose agar and actually suppressed switching on GlcNAc agar (Figure 3F). Overexpression of *TPK2*, however, caused a tenfold increase in the switching frequency on glucose agar over that of wild type cells and enhanced the frequency of switching by approximately 20% on GlcNAc agar (Figure 3F). These results suggested that in wild type cells, Tpk2 may function as the major downstream kinase in the GlcNAc induction pathway.

### Ras1/cAMP Pathway at 37°C

At 37°C, GlcNAc induction was impaired in the mutants *ras1/ras1* and *cdc35/cdc35* (supplemental Table S1) but to a lesser extent than at 25°C. High temperature induction, however, reinforced our conclusion that it is Tpk2 that plays the crucial role in wild type cells in transducing GlcNAc induction. Whereas at 25°C GlcNAc induction was unaffected in *tpk1/tpk1* and *tpk2/tpk2*, at 37°C it was reduced in the *tpk2/tpk2*, but not *tpk1/tpk1* (supplemental Table S1). These results supported our conclusion based on overexpression data (Figure 3B-E) that Tpk2 plays a role in transducing GlcNAc induction in wild type cells.

### The Role of *WOR1*

The *WOR1* (*TOS9*) locus has been demonstrated to regulate spontaneous white-opaque switching [5]–[7]. It has been proposed that a stochastic increase in *WOR1* expression above a threshold causes cells to switch from white to opaque, and that continued expression above that threshold maintains the opaque phenotype [5]–[7]. Wor1 has been shown to auto-induce at the level of transcription [5]–[7]. When activated, the cAMP pathway, which traditionally functions by cAMP-activation of protein kinase A, might increase the frequency of switching by phosphorylating either Wor1 or one of the several proteins that modulate *WOR1* function through transcriptional regulatory loops [8],[9] or chromatin modification [10]–[12]. Interestingly, Wor1 possesses a single consensus PKA phosphorylation motif, between amino acids 64 and 69 with a phosphorylatable threonine at amino acid 67 [5].

**Figure 4:**
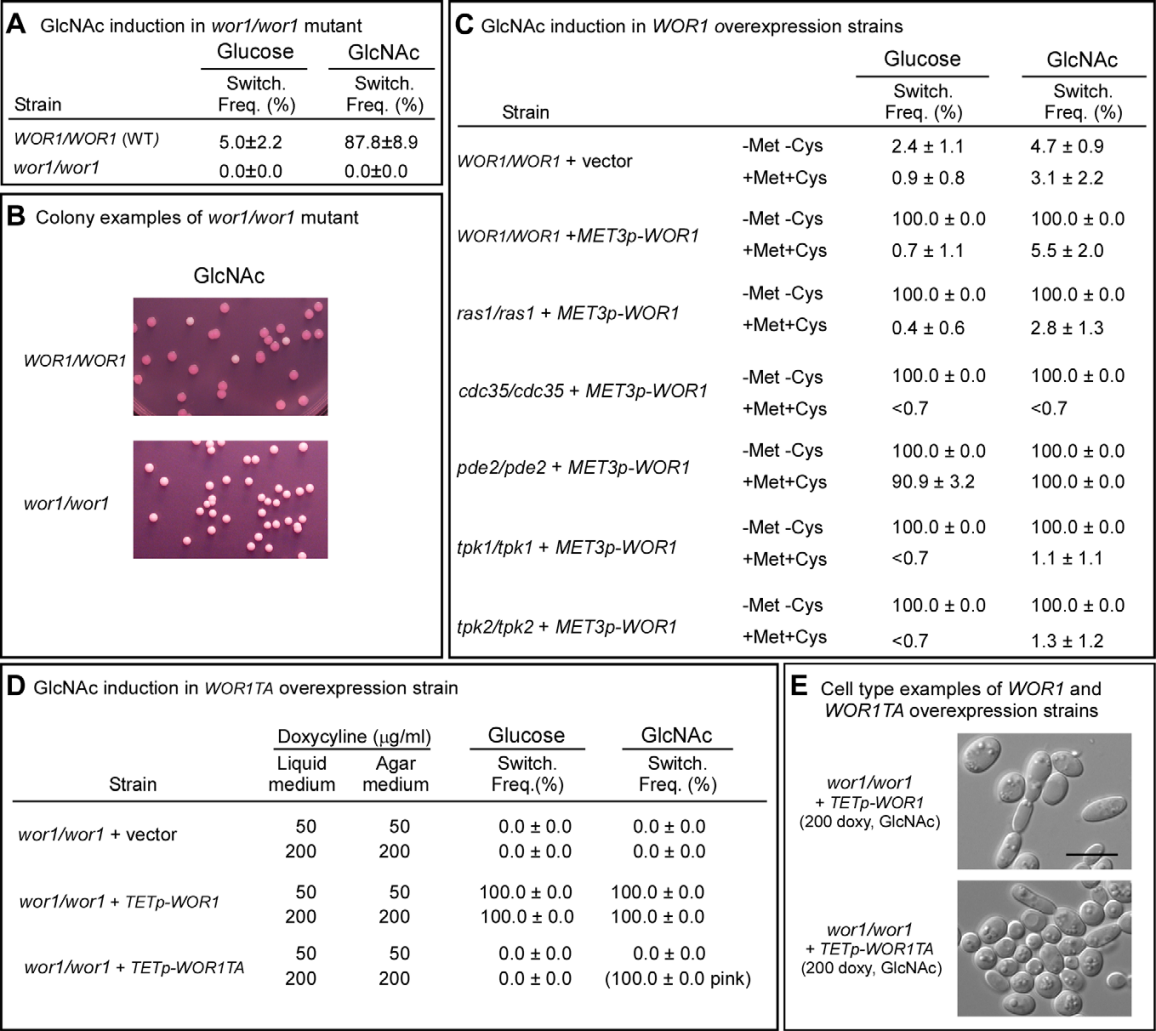
*WOR1*, the master switch locus, is essential for GlcNAc induction and involves phosphorylation. A. Switching frequency of the parental control and mutant *wor1/wor1* at 25°C. The number assessed colonies ranged between 250 and 450. B. Examples of colonies of the control and deletion mutant *wor1/wor1* grown at 25°C on GlcNAc agar. C. Switching frequencies of cells of the control *WOR1/WOR1*-vector and the overexpression derivatives *ras1/ras1*+*METp*-*WOR1*, *cdc35/cdc35*+*METp*-*WOR1*, *pde2/pde2*+*METp*-*WOR1*, *tpk1/tpk1*+*METp*-*WOR1* and *tpk2/tpk2*+*METp*-*WOR1* grown at 25°C in liquid glucose medium for 1 day and plated on either glucose or GlcNAc agar at 25°C under activating conditions (-Met, -Cys). The number assessed colonies ranged between 100 and 400. D. Switching frequencies of cells of the control *wor1/wor1*-vector, *wor1/wor1*+*TETp*-*WOR1* and *wor1/wor1*+*TETp*-*WOR1TA* grown in glucose liquid medium at 25°C and plated on glucose or GlcNAc agar containing 50 or 200 µg per ml of doxycycline, the *TET* inducer, at 25°C. The number of assessed colonies ranged between 100 and 300. E. Examples of cells from colonies of cells of *wor1/wor1*+*TETp*-*WOR1* and *wor1/wor1*+*TETp*-*WOR1TA* grown in glucose liquid medium at 25°C and plated on GlcNAc agar containing 200 µg of doxycycline at 25°C.

To test whether Wor1 was essential for GlcNAc-activated switching, white cells of the parental strain (WT) and the *WOR1* deletion mutant *wor1/wor1* were grown at 25°C to saturation phase (seven days) in liquid glucose medium and then plated on nutrient agar containing glucose or GlcNAc at 25°C. The *wor1/wor1* mutant did not switch on either glucose or GlcNAc agar (Figure 4A, B). Neither a single opaque colony or opaque sector was observed among more than 1,000 colonies. This was also true at 37°C (data not shown).

We then tested whether overexpression of *WOR1* drove the phenotype to opaque in the *ras1/ras1*, *pde2/pde2*, *cdc35/cdc35*, *tpk1/tpk1* and *tpk2/tpk2* mutants by transforming these mutants with a construct in which *WOR1* was under the regulation of the inducible *MET3* promoter [33]. In the activated state, 100% of white cells of all five strains, when plated on either glucose or GlcNAc agar at 25°C, switched to opaque (Figure 4C). These results demonstrated that *WOR1* is essential for the induction of switching by GlcNAc and that it plays a role downstream of the Ras1/cAMP pathway.

To test whether threonine phosphorylation is necessary for Wor1 function, the homozygous deletion mutant *wor1/wor1* was transformed with the *WOR1TA* construct, in which the phosphorylatable threonine 67 residue was replaced with the nonphosphorylatable amino acid alanine and the construct placed under the control of the inducible tetracycline promoter (*TETp*) to generate strain *wor1/wor1*+*TETp*-*WOR1TA*. A control strain *wor1/wor1*+*TETp*-*WOR1*, was generated in which the mutant *wor1/wor1* was transformed with a construct containing the native *WOR1* ORF under the regulation of the tetracycline promoter. A second control strain, *wor1/wor1*+vector, was also generated in which *wor1/wor1* was transformed with the vector lacking a *WOR1* derivative. White cells of the three test strains were grown in liquid glucose medium at 25°C to saturation phase (five days) and then plated on glucose or GlcNAc agar at 25°C. Both the liquid and agar media contained either 50 or 200 µg per ml of the tetracycline analog doxycycline, which had been shown to induce submaximal and maximal levels of expression, respectively [39]. When native *WOR1* was overexpressed both in glucose liquid medium and on glucose agar, the switching frequency was 100% at both 50 and 200 µg per ml of doxycycline (Figure 4D). When *WOR1TA* was overexpressed in both liquid glucose medium and then after plating on glucose agar at either 50 or 200 µg per ml of doxycycline, the frequency of switching was zero percent (Figure 4D). When native *WOR1* was overexpressed in both GlcNAc liquid medium and then after plating on GlcNAc agar at 50 and 200 µg per ml of doxycycline, 100% of the colonies underwent switching (Figure 4D). At 200 µg per ml of doxycycline, over 70% of the cells in the opaque colonies exhibited the elongate opaque cell phenotype (Figure 4E). When *WOR1TA* was overexpressed in both GlcNAc liquid medium and then after plating on GlcNAc agar at 50 µg per ml of doxycycline, 0% of the colonies exhibited switching (Figure 4D). However, when *WOR1TA* was overexpressed in GlcNAc media at 200 µg per ml of doxycycline, 100% of the colonies were light pink (Figure 4D). Microscopic analysis revealed that 10% of the cells in these pink colonies exhibited the elongate opaque phenotype (Figure 4E). These results suggested that expression of the unphosphorylatable derivative of Wor1, Wor1TA, was capable of inducing switching, but with a 10-fold reduction in efficiency.

**Figure 5:**
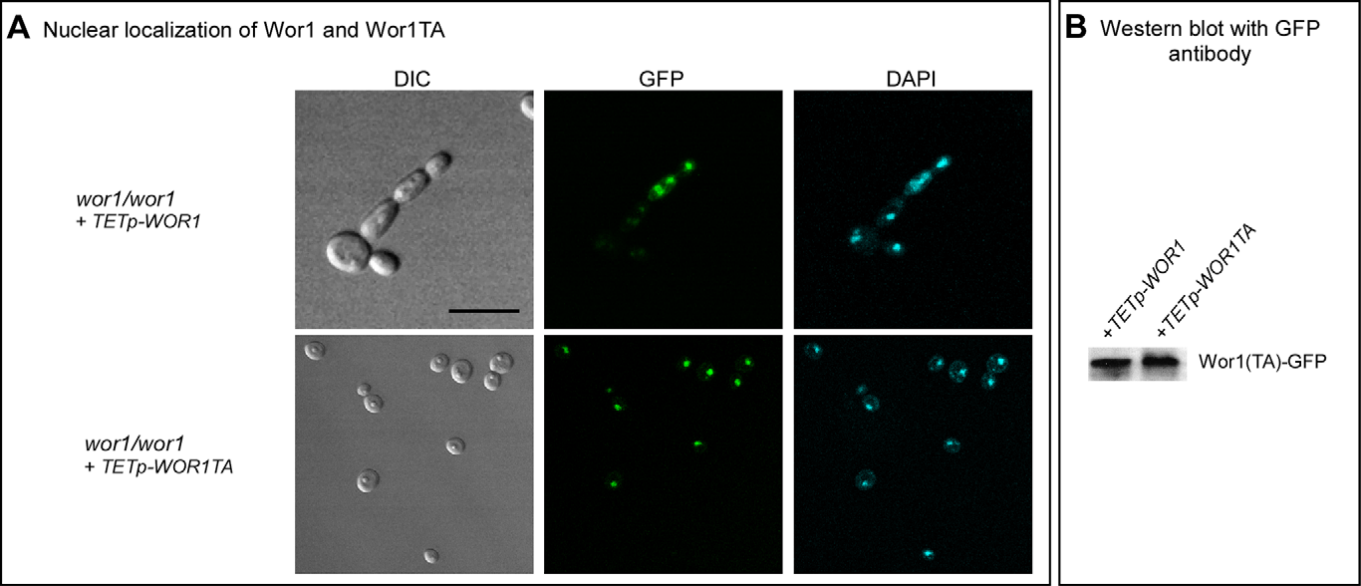
Wor1 localizes to the nucleus in the phosphorylated and unphosphorylated state. A. GFP fluorescence of Wor1 and Wor1TA, in parallel with DAPI staining of nuclei, of strains *wor1/wor1*+*TETp*-*WOR1* and *wor1/wor1*+*TETp*-*WOR1TA*, respectively. Cells were grown in glucose liquid medium containing 200 µg per ml of doxycycline at 25°C. B. Western blot analysis of Wor1 and Wor1TA using anti-GFP antibody in strains *wor1/wor1*+*TETp*-*WOR1* and *wor1/wor1*+*TETp*-*WOR1TA*. Protein extracts were derived from cells grown at 25°C in glucose liquid medium containing 200 µg per ml of doxycycline.

Because *WOR1* and *WOR1TA* were fused in frame with GFP in the overexpression mutants, we used confocal microscopy to test whether Wor1TA localized normally to the nucleus and was expressed at the same level as Wor1. Both Wor1 and Wor1TA localized to the nucleus of a majority of cells of the overexpression mutants treated with doxycycline, as demonstrated by overlapping GFP fluorescence and staining with DAPI, a DNA indicator (Figure 5A). Moreover, GFP fluorescence of nuclei was qualitatively comparable for Wor1-GFP and Wor1TA-GFP (Figure 5A). These results demonstrated that although the replacement of threonine with alanine caused a dramatic decrease in its capacity to support switching, it did not affect nuclear localization or cause a decrease in the transcript level. The levels of the Wor1 and Wor1TA protein were then compared by western blot analysis using anti-GFP antibody. The levels of Wor1 and Wor1TA expressed in white cells of strains *wor1/wor1*+*TETp*-*WOR1* and *wor1/wor1*+*TETp*-*WOR1TA*, respectively, treated with 200 µg per ml of doxycycline were similar (Figure 5B). These results indicate that the decrease in Wor1 function resulting from the replacement of threonine with alanine in the PKA consensus motif of Wor1 was due to a decrease in function, rather than to a decrease in the level of the Wor1 protein or mis-localization.

### Low CO_2_ Enhances GlcNAc Induction

We had demonstrated that 1% CO_2_ induced switching submaximally and that at this concentration induction was dependent primarily upon the Ras1/cAMP signal transduction pathway [18]. We have shown here that GlcNAc induction was also submaximal when cells were grown for only two days in glucose liquid medium to mid-log phase (Figure 1C, D). We therefore tested whether cells growing at 25 or 37°C in a suboptimal concentration of CO_2_ (1%) and for a suboptimal period of time in glucose liquid medium enhanced GlcNAc induction. White cells of an **a**/**a** and an α/α strain were first grown at 25°C in glucose liquid medium in air for two days and then plated on either glucose or GlcNAc agar either in air or in air containing 1% CO_2_ at 25 or 37°C. On glucose agar in 1% CO_2_ at both temperatures, cells of the **a**/**a** and α/α strains exhibited switching frequencies that were significantly higher than in air alone (Table 1). When plated on GlcNAc agar in air at 25°C, the respective frequencies of the two strains were 26.7±1.2% and 22.4±3.3%, but at 37°C, they were 98.9±2% and 99.7±0.6%. However, when plated on GlcNAc agar in 1% CO_2_, the switching frequency at the two temperatures were 99 to 100% in both strains (Table 1). These results indicate synergy for CO_2_ and GlcNAc induction, and enhancement by physiological temperature.

## Discussion

Recently, high CO_2_ was demonstrated to be a potent inducer of the white-opaque transition at 37°C [18]. The high concentrations of CO_2_ that induce and maintain the opaque phenotype at 37°C are found in select host tissues and in the gastrointestinal tract [40],[41]. The main source of CO_2_ in the gastrointestinal tract is the result of metabolism by colonic bacteria [40]. Here, we demonstrate GlcNAc, also a product primarily of bacteria that cohabit the host gastrointestinal tract with *C. albicans* [21]–[23],[42] represents a second potent inducer of the white to opaque transition.

By mutational analyses, we have found that the components of the Ras1/cAMP pathway, Ras1, Cdc35, Pde2 and PKA (Tpk1, Tpk2), mediate the major portion of GlcNAc induction. Our results also indicate that switching in glucose medium, which is at a far lower frequency than that in GlcNAc medium, is mediated in part by the Ras1/cAMP pathway. The low level of induction in deletion mutants of the Ras1/cAMP pathway is still approximately ten-fold higher than the level caused by glucose in wild type cells. Because the pathway that transduces high CO_2_ induction is also Ras1/cAMP-independent and unidentified, the possibility must be entertained that it may be the same Ras1/cAMP-independent pathway that mediates the minor portion of GlcNAc induction.

Our results, especially those at 37°C, suggest that the PKA isoform Tpk2 functions as the major downstream target of cAMP in the GlcNAc response pathway. Tpk1 appears to be capable of substituting for Tpk2 in the mutant *tpk2/tpk2*, but when overexpressed, inhibits GlcNAc-induced switching. One possible explanation is that the two Tpk isoforms play inhibiting and stimulating roles, respectively, in the white to opaque transition, especially in light of the fact that Efg1, a negative regulator of Wor1 [8], contains a PKA phosphorylation site. Tpk isoforms have been found to play different roles in the same regulatory networks in other systems. In *S. cerevisiae*, while Tpk2 activates filamentation downstream of cAMP in the glucose induced pathway, Tpk1 and Tpk3 inhibit filamentation by a feedback loop [36]. In addition, in the pheromone response pathway of *C. albicans*, the downstream MAP kinases Cek1 and Cek2 also play both distinct and overlapping roles [43], suggesting a general pattern of functional complexity of downstream protein kinase isoforms in signal transduction pathways.

Mutant analysis revealed that both the major and minor GlcNAc induction pathways required the master switch locus *WOR1*. Because cAMP activates PKA, we considered the possibility that Wor1, which contains one conserved PKA phosphorylation motif between amino acids 64 and 69, might have to be phosphorylated to function in the switch event. By converting the single threonine residue at that site to alanine, GlcNAc induction was impaired dramatically, indicating that phosphorylation of threonine 67 of Wor1 is necessary for maximum induction by GlcNAc. The observation that GlcNAc induction was completely blocked in the *wor1/wor1* mutant, but only impaired in the *wor1/wor1*+*TETp*-*WOR1TA* mutant suggested that the constitutively unphosphorylated form of Wor1 was still functional, but at far lower efficiency than the phosphorylated form. In *Schizosaccharomyces pombe*, the gluconate transport inducer 1 (Gti1), an ortholog of Wor1, also harbors one conserved PKA phosphorylation motif between amino acids 65 and 70, and conversion of the single threonine residue at that site to alanine causes severe impairment of function [44]. As is the case in *C. albicans*, Pka1, which is the only PKA in *S. pombe*, is involved in the regulation of Gtil. Given that in *C. albicans*, Wor1 has one conserved PKA phosphorylation site that must be phosphorylated to attain the major portion of GlcNAc induction, and that Tpk2 appears to be the downstream PKA involved in GlcNAc induction, it seems reasonable to suggest that GlcNAc induction may involve the direct phosphorylation of Wor1 by Tpk2, but that remains to be demonstrated.

**Figure 6:**
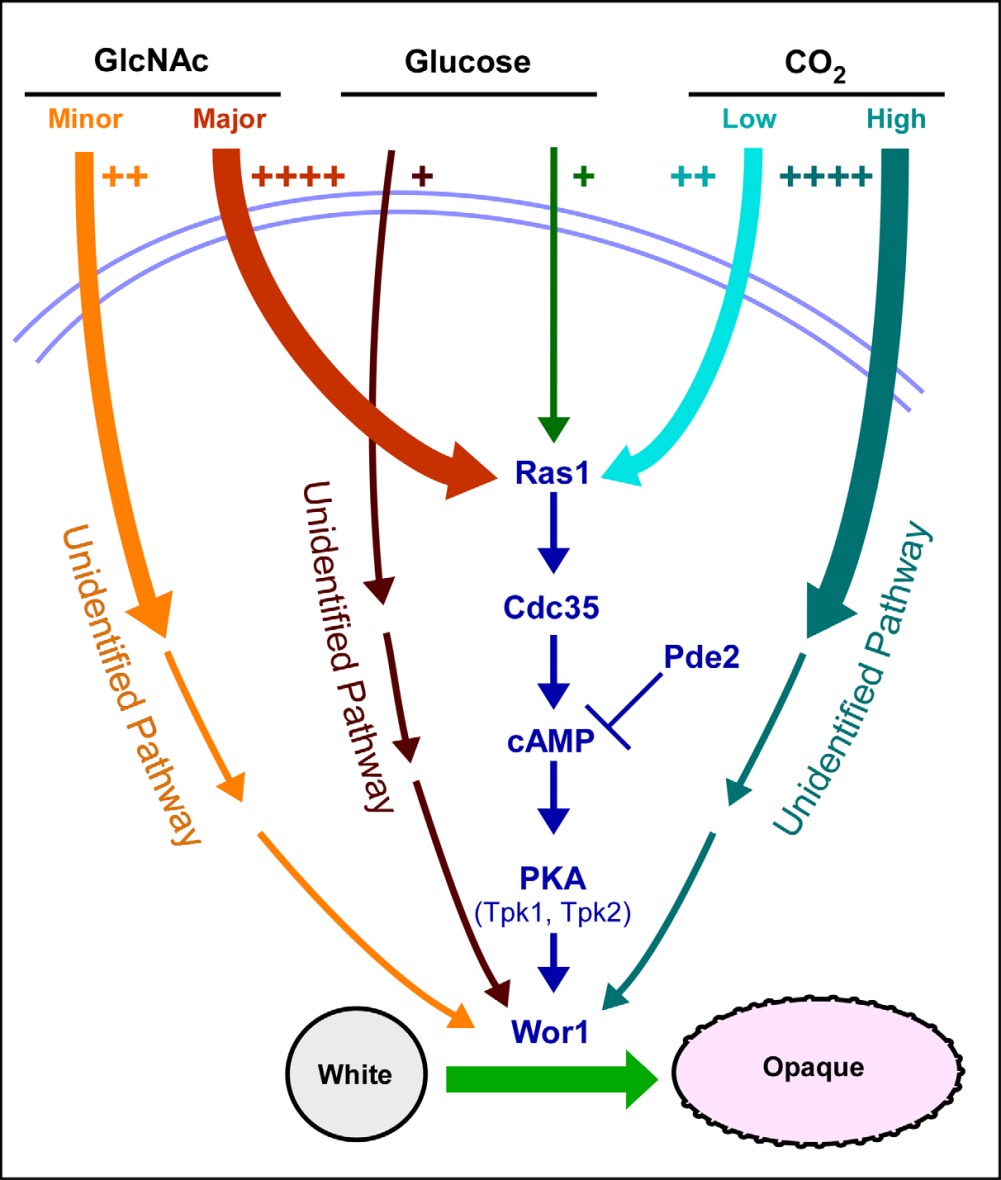
A model of the regulatory circuitry involved in the induction of the white to opaque switch. The number of plus signs and the thickness of initial pathway arrows reflect the degree of induction. Note that we have all pathways converging on the master switch gene *WOR1* because of their dependency on that gene.

The Ras1/cAMP-dependent pathway has been found to be the predominant one for GlcNAc induction and the minor one for low level CO_2_ induction [18] (Figure 6). An unidentified pathway has been found to be the predominant one for CO_2_ induction [18] and an unidentified pathway has the minor one for GlcNAc induction (Figure 6). Our data further suggest that glucose represents a weak but significant inducer of switching that also functions through both a Ras1/cAMP-dependent pathway and a Ras1/cAMP-independent pathway, the latter again unidentified (Figure 6). The fact that each inducer functions not only through the Ras1/cAMP pathway, but also through an unidentified pathway, leaves open the possibility that the Ras1/cAMP-independent pathway may also be common to all three inducers. We demonstrated previously that both the Ras1/cAMP-dependent and -independent pathways for CO_2_ induction are dependent on Wor1 [18], and we have demonstrated here that the dependent and independent pathways for G1cNAc and glucose induction are also dependent on Wor1. We have demonstrated that only the major portion of G1cNAc induction, which is transduced by the Ras1/cAMP pathway, requires the phosphorylated form of Wor1.

The induction of switching by environmental cues shares several characteristics with that of filamentation. First, both CO_2_ and GlcNAc induce filamentation [19],[20],[24] as they do switching. Second, the Ras1/cAMP pathway has been demonstrated to play a role in the induction of filamentation by CO_2_ [20], as it does in switching. The Ras1/cAMP pathway has also been demonstrated to play a role in the induction of filamentation in *S. cerevisiae* [36],[45], suggesting that the pathways regulating of filamentation represent an ancestral process conserved in the evolution of both the *Candida* and *Saccharomyces* groups of the hemiascomycetes. Several characteristics of the opaque phenotype are shared with hyphae, including an elongate shape, a prominent vacuole and cell surface antigens [46],[47]. Because white-opaque switching is a specific and unique characteristic of *C. albicans* and the closely related species *Candida dubliniensis* [48], it represents a newly evolved developmental process, in contrast to filamentation.

Switching of *C. albicans* from white to opaque at physiological temperature can therefore be influenced by two factors in the gastrointestinal tract that result primarily from gastrointestinal bacteria: high CO_2_ [18] and free GlcNAc. In host tissue, high CO_2_ is the result of metabolism by the host, but in the gastrointestinal tract, it is the product of bacterial metabolism [40],[41]. GlcNAc in the gastrointestinal tract is also a product primarily of gastrointestinal tract bacteria, but also to a lesser extent of host goblet cells [41]. Hence, bacteria of the gastrointestinal tract produce two potent inducers of the white to opaque transition, a prerequisite for mating between **a**/**a** and α/α cells [4]. These results suggest that two developmental programs of *C. albicans*, filamentation and switching, have evolved to respond to signals originating from bacterial co-members of the gastrointestinal microbiota.

## Materials and Methods

### Strain Maintenance and Growth

The strains of *C. albicans* used in this study are listed in supplemental Table S2. For routine growth, modified Lee's medium without methionine was used [31], unless stated otherwise. For repression of *MET3* promoter-controlled gene expression, 2.5 mM methionine and 2.5 mM cysteine were added to the medium. For GlcNAc induction, the carbon source glucose was replaced with GlcNAc (1.25% w/v) in nutrient medium. Here, agar containing Lee's medium, in which glucose was the carbon source, was referred to as glucose agar and Lee's medium containing GlcNAc as a carbon source was referred to as GlcNAc agar. Agar cultures were grown at a density of 80–120 colonies per 85 mm plate. Phloxine B was added to nutrient agar for opaque colony staining [46].

### Construction of *C. albicans* Deletion Mutants

The *PDE2* gene was disrupted using a modified Ura-blaster method [49]. Two long primers (PDE2-5DR, PDE2-3DR), each containing a different 60 nucleotide sequence homologous to the gene *PDE2*, were used for PCR amplification (supplemental Table S3). pDDB57, which contains the recyclable URA3-dpl200 marker, was used as template. The PCR product was transformed into WUM5A, a WO-1 derivative [50]. Transformants were grown on selective synthetic defined (SD) medium SD-Ura agar plates. To delete the second allele of *PDE2*, the PCR product was transformed into a spontaneous Ura- derivative of *PDE2/pde2* obtained from SD agar containing 5-fluoro-orotic acid. *pde2/pde2* null mutants were selected from SD-Ura agar plates and confirmed by PCR.

*TPK1* and *TPK2* were deleted by a PCR product-directed disruption protocol, as described in [18]. Briefly, the *HIS1* and *ARG4* markers were amplified by PCR from pGEM-HIS1 and pRS-ARG4-SpeI, respectively. The oligonucleotide pairs TPK1-5DR, TPK1-3DR; TPK2-5DR, TPK2-3DR (supplemental Table S3) were used for PCR amplification. The *HIS1* and *ARG4* markers were sequentially transformed into the host strains GH1013 [51], and heterozygous mutants. The null mutants were selected on SD-His-Arg plates and confirmed by PCR.

### Construction of Plasmids

The primers used for plasmid constructions are listed in supplemental Table S3. To generate pMET-RAS1V13, the *RAS1* ORF containing a mutation at the thirteenth amino acid (glycine to valine mutation) was amplified from pQF145.2 [25] by using primers including *Pst*I and *Sph*I sites, and then cloned into pCaEXP [33]. pMET-CDC35 was constructed by inserting the *Bam*HI-*Sph*I digested *CDC35* ORF fragment into pCaEXP. To generate pMET-PDE2, the *PDE2* ORF was amplified from CAI4 genomic DNA by using the primers PDE2F and PDE2R that contained *Bam*HI and *Sph*I sites (supplemental Table S3) and the *PDE2* ORF was cloned into pCaEXP. To generate pACT-TPK1, the *TPK1* ORF was amplified from CAI4 genomic DNA by using the primers TPK1F and TPK1R that included *Eco*RV and *Hind*III sites (supplemental Table S3), and the *TPK1* ORF was cloned into pACT1 [18]. The *TPK2* ORF was amplified from CAI4 genomic DNA by using primers TPK2F and TPK2R (supplemental Table S3). To generate pACT-TPK2, the PCR product was digested by *Hind*III and cloned into *Eco*RV/*Hind*III-digested pACT1. pNIM-WOR1 was constructed by inserting a *Sal*I digested PCR fragment of *WOR1* into the *Sal*I site of pNIM1 [52]. The *WOR1* ORF was amplified from CAI4 genomic DNA. The primers WOR1salF and WOR1salR were used for PCR amplification (supplemental Table S3). To generate a mutation in which threonine 67 is replaced with alanine in the *WOR1* gene, a two-step PCR method [53] was used with slight modification. The primers WOR1TAF and WOR1TAR (supplemental Table S3) were used to generate site-directed mutation. The second-round PCR product was digested with *Sal*I and subcloned into the *Sal*I site of pNIM1. The resulting plasmid was referred to as pNIM-WOR1TA. The correct direction of *WOR1* ORF and *WOR1TA* fragment in pNIM1 was confirmed by sequencing.

### Regular White-Opaque Switching Assay (from Agar to Agar)

White-opaque switching on agar was analyzed as described previously [15]. Briefly, strains were first grown on agar containing supplemented Lee's medium for 6 days at 25°C. Colonies were then replated onto plates containing supplemented Lee's medium [31]. These plates were then incubated at 25°C for five days, and the proportion of colonies exhibiting different phenotypes counted.

### White to Opaque Switching of Cells in Different Growth Phases

White colonies were inoculated into a test tube containing 1 ml of supplemented Lee's medium [31] with glucose as carbon source and incubated at 25°C. The overnight culture was diluted (to 2×10^5^ cells/ml) in 20 ml of fresh medium with glucose as the carbon source and incubated at 22°C in a shaker. Aliquots were taken out at different time points, diluted and plated onto both glucose agar and GlcNAc agar plates (Figure 1A). The plates were then incubated at 25°C for five days, and the colonies exhibiting different colony phenotypes counted. For the experiments performed at 37°C, plates were cultured at both 25 and 37°C.

### Western Blot Analysis

Cells from liquid cultures were spun down following doxycycline treatment for 12 hours. Total protein extract was obtained using a bead beater in lysis buffer that contained 50 mM Tris-HCl, 100 mM NaCl, 5 mM MgCl_2_, 1 mM DTT, 1 mM EDTA, 1 mM EGTA, 0.1% Tween-20, and 5% glycerol, supplemented with a protease inhibitor cocktail (Sigma-Aldrich, St Louis, MO) and 1 mM phenyl-methylsulphonyl fluoride. An equal amount of total protein from each sample was then subjected to protein G beads (Active Motif, Carlsbad, California) for pre-clearing, followed by immuno-precipitation (IP) using rabbit GFP antibody-conjugated agarose beads (Santa Cruz Biotechnology, Santa Cruz, California). IP protein samples were subjected to SDS-PAGE (8% polyacrylamide) electrophoresis. After electrophoresis, the SDS-PAGE protein gel was transferred to a PVDF membrane (Immobilon-P, Millipore Corporation, Bedford, MA), blocked for 1 h in 3% non-fat dry milk in TBS-T (20 mM Tris-HCl, pH 7.5, 150 mM NaCl, 0.05% Tween-20), and then incubated with rabbit polyclonal GFP antibody (Santa Cruz Biotechnology, Santa Cruz, CA) overnight at 4°C [54]. After washing six times in TBS-T, the proteins on the membrane were detected with horseradish peroxidase-labelled goat anti-rabbit IgG (Promega, Madison, WI) and SuperSignal West Pico Chemiluminescent Substrate (Pierce, Rockford, IL).

### Co-localization of GFP-Wor1p and Nuclei

Cells expressing tetracycline (doxycycline)-inducible GFP-labeled Wor1p were grown to midlog phase in the presence of 50 µg/ml doxycycline (Sigma-Aldrich, St Louis, MO, USA), harvested and simultaneously permeabilized and the nuclei labeled with 4′,6′-Diamidino-2-phenylindole (DAPI, Invitrogen, Inc.) by incubating them for 10 min at room temperature in the dark in a solution containing 5 µg/ml DAPI in 1 M Sorbitol, 0.1% Saponin, 150 mM NaCl and 20 mM Tris buffer, pH 7.4, followed by a 15–20 min incubation period on ice. Without washing, the cells were imaged using a Bio-Rad Radiance 2100 MP multi-photon microscope (Bio-Rad, Hermel, Hamstead, UK). Cells were excited at 780 nm by a Mai-Tai laser (Spectra- Physics, Newport Corp., Mountain View, CA) and three channel emission images (GFP, DAPI and transmitted) were gathered using a sequential 2.0 µm Z-series, gathered at 0.2 µm intervals to include the entire cell nucleus. GFP and DAPI images were visualized as Z-series projections. Transmitted images were a single scan at the focal plane selected from the Z-series.

## Supporting Information

Table S1 Ras1/cAMP pathway is required for GlcNAc induction of white to opaque switching at 37°C.
(0.03 MB DOC)

Table S2 Strains used in this study.
(0.04 MB DOC)

Table S3 Primers used in this study.
(0.04 MB DOC)
