# Correction: N-Acetylglucosamine Induces White to Opaque Switching, a Mating Prerequisite in *Candida albicans*

**DOI:** 10.1371/annotation/ed4d1473-cf80-4e85-ad9d-7a390be260f6

**Published:** 2010-03-26

**Authors:** Guanghua Huang, Song Yi, Nidhi Sahni, Karla J. Daniels, Thyagarajan Srikantha, David R. Soll

There were errors in the Author Contributions. The correct contributions are: Conceived and designed the experiments: GH DRS. Performed the experiments: GH SY NS KJD TS. Analyzed the data: GH DRS. Wrote the paper: GH DRS. Made the figures: GH KJD.

