# Supplementary material for: N-Acetylglucosamine Induces White to Opaque Switching, a Mating Prerequisite in Candida albicans
Source: PLoS Pathog. 2010 Mar 12;6(3):e1000806. doi: 10.1371/journal.ppat.1000806 (PMC2837409; doi:10.1371/journal.ppat.1000806)
Supplement: Table S1 — Ras1/cAMP pathway is required for GlcNAc induction of white to opaque switching at 37°C. (0.03 MB DOC) [file ppat.1000806.s001.doc]

**Supplemental Table 1. Ras1/cAMP pathway is required for GlcNAc induction of white to opaque switching at 37 °C.**

| Strain | Glucose | GlcNAc |
| --- | --- | --- |
|  | Switching frequency (%) | Switching frequency (%) |
| WT(GH1060) | <0.4 | 99.4±0.5 |
| *ras1/ras1* | <0.3 | 1.4±0.2 |
| *cdc35/cdc35* | <0.3 | <0.3 |
| *tpk1/tpk1* | <0.3 | 95.8±1.0 |
| *tpk2/tpk2* | <0.3 | 13.2±4.0 |
| *pde2/pde2* | <0.5 | 100.0±0.0 |

As described in Figure 1A, white cells were cultured for 48 hours in glucose liquid medium at 25 °C, then plated onto glucose or GlcNAc agar, and incubated at 37 °C in air. Total colonies analyzed in each strain and condition varied between 200 and 400.
