# Supplementary material for: N-Acetylglucosamine Induces White to Opaque Switching, a Mating Prerequisite in Candida albicans
Source: PLoS Pathog. 2010 Mar 12;6(3):e1000806. doi: 10.1371/journal.ppat.1000806 (PMC2837409; doi:10.1371/journal.ppat.1000806)
Supplement: Table S2 — Strains used in this study. (0.04 MB DOC) [file ppat.1000806.s002.doc]

**Supplemental Table S2. Strains used in this study**

| Strain | Parent strain | Genotype | Reference |
| --- | --- | --- | --- |
| 5314**a** | SC5314 | *MTL***a/a** | **[18]** |
| 5314α | SC5314 | *MTL*α/α | **[18]** |
| WO-1 |  | *MTL*α*/*α | **[15]** |
| WUM5A | WO-1 | *MTL*α*/*α *ura3-1::FRT/ura3-2::FRT* | **[51]** |
| TOHO3 | WUM5A | *MTL*α*/*α *ura3-1::FRT/ura3-2::FRT wor1::FRT/wor1::FRT* | **[6]** |
| GH1079 | WUM5A | *MTL*α*/*α *ura3-1::FRT/ura3-2::FRT pde2::dpl200/pde2::URA3-dpl200* | **This study** |
| GH1060 | CAI4 | *MTL*α*/mtl***a***:: dpl200 ura3:imm434/ura3::imm434* | **[18]** |
| GH1120 | CAN52 | *MTL*α*/mtl***a***::dpl200* *ras1::hisG/ras1::hph ura3::imm434/ura3::imm434* | **[18]** |
| GH1013 | BWP17 | *MTL***a/a** *ura3::imm434/ura3::imm434 his1::hisG/his1::hisG arg4::hisG/arg4::hisG* | **[18]** |
| GH1109 | GH1013 | *MTL***a**/**a** *ura3::imm434/ura3::imm434 his1::hisG/his1::hisG arg4::hisG/arg4::hisG cdc35::ARG4/cdc35::HIS1* | **[18]** |
| GH1126 | GH1013 | *MTL***a**/**a** *ura3::imm434/ura3::imm434 his1::hisG/his1::hisG arg4::hisG/arg4::hisG tpk1::ARG4/tpk1::HIS1* | **This study** |
| GH1148 | GH1013 | *MTL***a**/**a** *ura3::imm434/ura3::imm434 his1::hisG/his1::hisG arg4::hisG/arg4::hisG tpk2::ARG4/tpk2::HIS1* | **This study** |
