# Supplementary material for: N-Acetylglucosamine Induces White to Opaque Switching, a Mating Prerequisite in Candida albicans
Source: PLoS Pathog. 2010 Mar 12;6(3):e1000806. doi: 10.1371/journal.ppat.1000806 (PMC2837409; doi:10.1371/journal.ppat.1000806)
Supplement: Table S3 — Primers used in this study. (0.04 MB DOC) [file ppat.1000806.s003.doc]

**Supplemental Table S3. Primers used in this study**

| Name | Sequence | Purpose and features |
| --- | --- | --- |
| WOR1F | ttaccaagatctATGTCTAATTCAAGTATAGTCCCTAC | pMET-WOR1 |
| WOR1R | attacccccgggCTAAGTACCGGTGTAATACGACCC |  |
| PDE2F | acagattGGATCCATGGCAGAAGTATTATCATTGGTTG | pMET-PDE2 |
| PDE2R | acagattGCATGCATTGTTATTTCTTTGCTCTTTCCAACC |  |
| RAS1V13F | acgaatactgcagatgttgagagaatataaattag | pMET-RAS1V13 |
| RAS1V13R | acgaatagcatgctcaaacaataacacaacatcc |  |
| TPK1F | tcatcaaGATATCATGACATCCATGGAACCAGCAGAC | pACT-TPK1 |
| TPK1R | tcattagaagcttAAAAGTCCTGGAATTGATCACGATA |  |
| TPK2F | ATGGTGAATCTTTTAAAGAAACTTC | pACT-TPK2 |
| TPK2R | tcattagaagcttCAAAAGTCAAGGAAATACAGAGC |  |
| PDE2-5DR | GCAGAAGTATTATCATTGGTTGACCTCGAGATTCCTCAAGTCACTGATAAGTATTATAAAGTTTTCCCAGTCACGACGTT | *PDE2* KO |
| PDE2-3DR | CTTTGCTCTTTCCAACCAAAATAGTTTGTTTTCCATAATAATATCACAAGTATACTGCAATGTGGAATTGTGAGCGGATA |  |
| TPK1-5DR | CAATTAATATTATCATTGAATAATAGATACTTATAGCAGTTATAGTAGAATTTAATTTCTGTTTTCCCAGTCACGACGTT | *TPK1* KO |
| TPK1-3DR | CTATAAAACTAGTTATCATAATTAACATTGTTGTGCCAATAAATACAATTTTATTTTTACTGTGGAATTGTGAGCGGATA |  |
| TPK2-5DR | ACAAAGAAGATTAGACAAACAATCACCCACTCACACCTACTACTCACCCAATTTCCATTCGTTTTCCCAGTCACGACGTT | *TPK2* KO |
| TPK2-3DR | TCTTACAGTTACTATCGTTATTATTTAGTCATTTATTCATTTATGAAAGTTCATCTCCTCTGTGGAATTGTGAGCGGATA |  |
| WOR1-SalF | aatcttgtcgacATGTCTAATTCAAGTATAGTCCC | pNIM-WOR1 |
| WOR1-SalR | aatcttgtcgacaaAGTACCGGTGTAATACGACCC |  |
| WOR1TAF | GAATCAAAAGATGGGCAGATGGTATTTCATGG | WOR1 TA mutation |
| WOR1TAR | CCATGAAATACCATCTGCCCATCTTTTGATTC |  |
| CDC35F | acagattGGATCCAtgagttttttaaggagagataaatc | pMET-CDC35 |
| CDC35R | acagattGCATGCtatctatttaagttcattaactgttttc | pMET-CDC35 |
